# Virus–Host Protein–Protein Interactions between Human Papillomavirus 16 E6 A1 and D2/D3 Sub-Lineages: Variances and Similarities

**DOI:** 10.3390/ijms21217980

**Published:** 2020-10-27

**Authors:** Guillem Dayer, Mehran L. Masoom, Melissa Togtema, Ingeborg Zehbe

**Affiliations:** 1 Biology Department, Lakehead University, Thunder Bay, ON P7B 5E1, Canada; gdayer@lakeheadu.ca; 2 Thunder Bay Regional Health Research Institute, Probe Development and Biomarker Exploration, Thunder Bay, ON P7B 6V4, Canada; mlmasoom@lakeheadu.ca (M.L.M.); mtogtema@lakeheadu.ca (M.T.); 3 Northern Ontario School of Medicine, Lakehead University, Thunder Bay, ON P7B 5E1, Canada

**Keywords:** human papillomavirus, E6 oncoprotein, sub-lineages, carcinogenesis, interactome study, co-immunoprecipitation, mass spectrometry, metabolic pathways, proteomics

## Abstract

High-risk strains of human papillomavirus are causative agents for cervical and other mucosal cancers, with type 16 being the most frequent. Compared to the European Prototype (EP; A1), the Asian-American (AA; D2/D3) sub-lineage seems to have increased abilities to promote carcinogenesis. Here, we studied protein–protein interactions (PPIs) between host proteins and sub-lineages of the key transforming E6 protein. We transduced human keratinocyte with EP or AA E6 genes and co-immunoprecipitated E6 proteins along with interacting cellular proteins to detect virus–host binding partners. AAE6 and EPE6 may have unique PPIs with host cellular proteins, conferring gain or loss of function and resulting in varied abilities to promote carcinogenesis. Using liquid chromatography-mass spectrometry and stringent interactor selection criteria based on the number of peptides, we identified 25 candidates: 6 unique to AAE6 and EPE6, along with 13 E6 targets common to both. A novel approach based on pathway selection discovered 171 target proteins: 90 unique AAE6 and 61 unique EPE6 along with 20 common E6 targets. Interpretations were made using databases, such as UniProt, BioGRID, and Reactome. Detected E6 targets were differentially implicated in important hallmarks of cancer: deregulating Notch signaling, energetics and hypoxia, DNA replication and repair, and immune response.

## 1. Introduction

Human papillomaviruses (HPVs) are double-stranded DNA viruses that infect keratinocytes of skin and mucosal tissues [1]. Persistent infection with any of the high-risk (HR) types is implicated in almost every case of cervical cancer [2,3], as well as having an aetiological association with other anogenital and head and neck cancers [4]. HPV16 (a member of Alphapapillomavirus-9 species) is one of the most common HR types [3].

Intracellular oncoproteins (E6 and E7) play an important role in the immortalization and malignant transformation of HPV-infected cells [5]. Even though both proteins are important for carcinogenesis, studying them independently allows for identification of pathways targeted by each oncoprotein. For the past 30 years, researchers have extensively studied how the E6 protein deregulates and transforms host cells [6,7]. HPV16 E6 confers viral persistence by downregulating type I interferons (IFNs) alpha, beta [8,9], kappa [10,11,12], as well as type II IFN gamma [13]. E6 prevents retention of Langerhans cells in infected epithelia through the downregulation of E-cadherin [14]. E6 induces cellular immortalization through simultaneous upregulation of human telomerase reverse transcriptase (hTERT), the catalytic subunit of the enzyme telomerase [15,16,17,18], and degradation of the tumour suppressor protein P53 [19,20]. A transformed cellular phenotype is promoted when the C-terminus of E6 binds to PDZ domain-containing proteins, such as membrane-associated guanylate kinase (MAGI)-1 [21], MAGI-2, MAGI-3 [22], hScribble [23], and hDlg [24,25], disrupting cell–cell signalling, cellular adhesion, and cell polarity.

**Figure 1:**
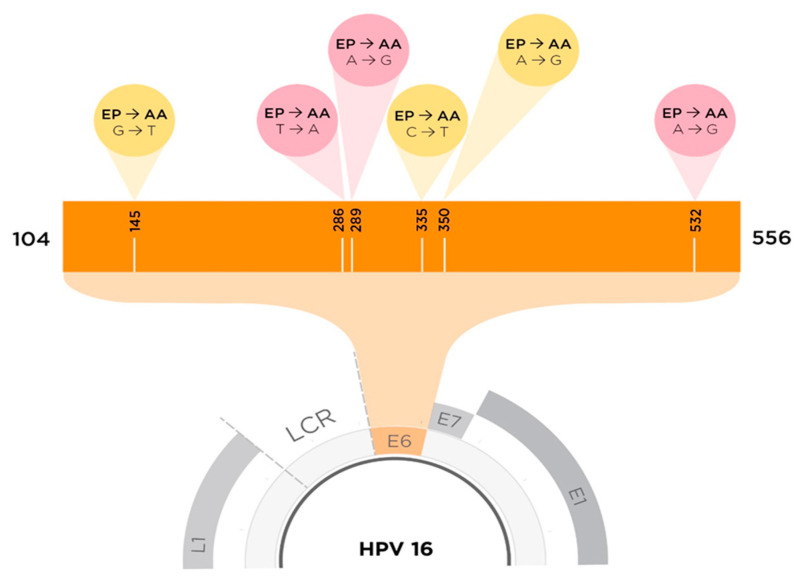
To scale depiction of HPV16 E6 SNPs between AAE6 and EPE6. The yellow cones indicate an SNP that results in an amino acid change (missense mutation), whereas the pink cones indicate no amino acid changes at that particular site (nonsense mutation). SNPs resulting in amino acid changes from EPE6 to AAE6 (Q14H, H78Y, and L83V) are found at nucleotide (NT) positions G145T, C335T, and T350G. SNPs that do not result in any change in amino acids are found at NT positions: T286A, A289G, and G532A [28].

HPV16 is comprised of four sub-lineages or “variants”: A (European prototype sub-lineages A1/A2/A3 and the Asian sub-lineage A4), B (African-1 sub-lineages B1/B2), C (African-2), and D (North American sub-lineage D1 and Asian-American sub-lineages D2/D3) [26,27]. We and other groups have been studying three HPV16 E6 protein variants (Figure 1): European prototype (EP), European-T350G (E-T350G: has an L83V single nucleotide polymorphism (SNP) in the E6 protein), and Asian-American (AA: has Q14H/H78Y/L83V SNPs in the E6 protein) [28,29,30,31]. Epidemiologically, the AA variant is associated with a higher risk for developing high-grade cervical lesions and cervical cancer, as well as with an earlier cancer onset [32,33,34,35]. The epidemiological evidence surrounding the E-T350G variant is more complex, showing population-specific risk [36,37]. Our functional investigations demonstrated that EP, E-T350G, or AAE6 retrovirally transduced into primary human foreskin keratinocytes (PHFKS) is able to immortalize these cells even in the absence of E7 [36,38]. While E-T350G and AAE6 promoted quicker cell growth and an earlier escape from growth crises than EPE6, only AAE6-transduced cells were able to form viable colonies in soft agar and demonstrate the ability to migrate and become invasive. Similar results were seen when E7 was also present [39]. When keratinocytes were grown in 3-D raft cultures, E-T350G and AAE6 caused higher grade dysplasia with more severe abrogation of differentiation patterns than EPE6 [29,40]. We found that AAE6 elevated the levels of glycolytic enzymes related to the tumour cell hallmark Warburg effect [39] and that of hypoxia inducible factor 1 alpha (HIF-1α) [41], possibly contributing to AAE6′s enhanced proliferative abilities. The deregulation of HIF-1α was caused by AAE6′s ability to augment mitogen-activated protein kinase/extracellular-related kinase (MAPK/ERK) signaling. It has also been shown that E-T350G differentially affects the amount of MAPK and phosphoinositide 3-kinases (PI3K)/activated kinase tyrosine (AKT) signaling [42,43,44], binding to calcium-induced binding protein and hDlg [45] as well as E-cadherin [36].

To elucidate the mechanisms behind some of the above-mentioned functional observations, Zacapala-Gómez et al. (2016) [30] completed a global transcriptome study and found 387 differentially expressed genes in C33A cells and compared to C33A cells transfected with EPE6. The genes involved were related to cellular processes, such as adhesion, angiogenesis, apoptosis, differentiation, cell cycle, proliferation, transcription, and protein translation; specifically, they found over-representation of more than 1.5-fold for immunological processes for AAE6 compared to EPE6-transfected cells. Of course, the carcinogenic C33A cells may already have had underlying alterations in the expression of these pathways prior to the introduction of the E6 protein. A whole HPV16 genome sequencing study using 7116 pooled patient samples reported that the D2/3 (AA) as well as A3/4 sub-lineages show increased cervical cancer risk compared to A1 (EP of the current study) [46]. Thus, variant E6 proteins are not merely interacting in different amounts with the same set of host proteins. Instead, amino acid changes in E6 may allow it to bind a unique set of host proteins.

In this study, we explored the most likely mechanism for the described E6 functions: protein–protein interaction (PPI) between two key E6 variants and host cellular targets. We first surveyed the literature and summarized the findings of 50 previously reported HPV16 E6 interactors (Table S1). Notably, most, if not all, interaction experiments of the 50 known binders did not specify which HPV16 E6 sub-lineage was used other than “wildtype” or “prototype” with or without artificial mutants. Thus, we started filling this gap by investigating two common naturally occurring E6 variants of the A1 (EPE6) and D2/D3 (AAE6) sub-lineages, based on our hypothesis that the type of PPIs between E6 and host cell proteins affects the carcinogenic potential of the HPV16 variant. Our methodological approach used primary human foreskin keratinocytes—naturally targeted by HPV—as well as state-of-the-art liquid chromatography-MS for virus–host PPI analysis and regularly curated freely accessible databases. The obtained results will facilitate many future studies in HR HPV biology and may prove useful for anticancer treatment strategies targeting E6 [47].

## 2. Results

We performed two independent co-immunoprecipitation (co-IP) coupled to MS trials. After the selection process, we were left with *n* = 878 proteins (material and methods, Section 4.4; Tables S2 and S3). For our report, we firstly used the “Peptide Method” (Section 2.1) based on previous HPV-related publications [18,48], and secondly, employed a novel approach denoted the “Protein-Pathway Method” (Section 2.2).

### 2.1. Peptide Method: E6 Activities Relate to P53 Tumour Suppression, Hypoxia, Energetics, Chromosome Remodeling, and Innate Immunity

**Figure 2:**
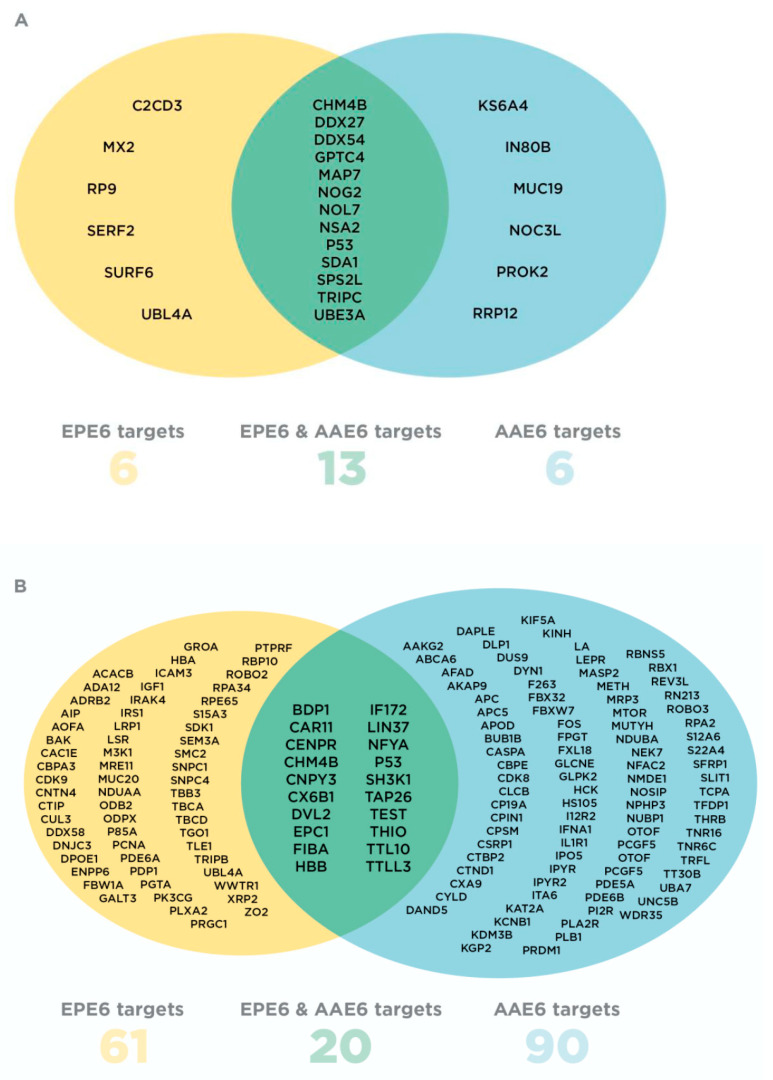
Venn diagrams representing AAE6 and EPE6 host cellular targets for both selection approaches: Peptide Method (**A**) and Protein-Pathway Method (**B**). EPE6-specific interactors are in yellow, AAE6-specific interactors are in blue and proteins common to both variants are in green. Protein names are abbreviated based on UniProt protein rather than gene nomenclature, enabling easy identification for searches and further information.

For the Peptide Method, two stringent rules were followed: any EPE6- or AAE6-targeted protein must have a sum of at least 2 peptides in either trial, independent of treatment, OR any EPE6- or AAE6-targeted protein must have at least 1 peptide in both trials, independent of treatment. After sorting the proteins with Excel, our peptide selection strategy left us with 25 candidates: 6 unique AAE6 and 6 unique EPE6-interacting proteins along with 13 common E6-interacting proteins between AAE6 and EPE6 (Figure 2A; Table S4). The 25 potential E6 interactors were screened for functions related to HPV16-related tumourigenesis and immune suppression, resulting in a short-list of 7 proteins. Three are targeted by AAE6: inositol (INO)80 complex subunit B (IN80B, Q9C086), ribosomal protein S6 kinase alpha-4 (KS6A4, O75676), prokineticin-2 (PROK2, Q9HC23), one by EPE6: interferon-induced GTP-binding protein (MX2, P20592), and three by both AAE6 and EPE6: E3 ubiquitin-protein ligase trip12 (TRIPC, Q14669), nucleolar GTP-binding protein 2 (NOG2, Q13823), charged multi-vesicular body protein 4b (CHM4B, Q9H444). To gain further insight into the functions of the 7 selected proteins, we subjected them to Reactome analysis to identify the pathways involving them.

#### 2.1.1. AAE6 Affects Chromosome Remodeling, Hypoxia, and Innate Immunity

IN80B, an ATPase, is part of the ATP-dependent INO80 remodeling complex containing at least 12 other proteins. The INO80 complex functions in transcriptional regulation, DNA replication and repair, telomere maintenance, and chromosome segregation [49]. In a mouse model of cervical cancer [50], INO80 was overexpressed and, when bound to the Nanog transcription start site, this transcription factor’s expression was enhanced. Blocking its interaction led to a decrease in proliferation. In addition, yeast 2 hybrid (Y2H) experiments indicated that IN80B interacts with HPV16 E7 and HPV5 E7 [51], suggesting that both E6 and E7 could alter the function of IN80B. Proximity label MS showed that the Myc proto-oncogene protein (MYC), a known target of E6 ([52], Table S1), also interacts with NOC3L and RRP12 ([53], Table S1). Notably, IN80B and RRP12 were present only in the AAE6 sample, NOC3L was targeted by both E6 variant proteins, whereas RP9 was unique to EPE6. Hence, even if EPE6 does not immunoprecipitate IN80B, it may alter its functions indirectly. Reactome analysis showed IN80B to be associated mainly with DNA damage and repair pathways and metabolism of proteins (Table S5).

KS6A4 was initially identified as mitogen- and stress-activated protein kinase 2, MSK2 (75% identical to MSK1), and is activated by the mitogen-activated protein kinase (MAPK)/extracellular signal-regulated kinase (ERK) or stress-activated protein kinase (SAPK)/p38 phosphorylation cascade [54], reviewed in [55]. MSK1 and 2 regulate the transcription factors cAMP response element-binding protein (CREB) and activating transcription factor 1 (ATF1) through phosphorylation [54]. MSKs were suggested to be negative regulators of the innate immune system as their regulation of CREB and ATF1 also controls the expression of interleukin-10 and dual-specificity protein phosphatase 1 [56]. They are further involved in chromatin remodeling by phosphorylating histone H3 and the high-mobility group chromosomal protein (HMG) 14 [57]. Chromatin remodelers are “essential for all DNA-dependent processes” [58], a fact that connects them with the above-described AAE6 interactor IN80B. MSK2 negatively regulates p53 in a kinase-independent manner through its interaction with P300 and its binding to the NOXA (DNA damage- and genotoxic stress-related) promoter [59]. Consistent with these inhibitory functions, MSK2 inhibits apoptotic processes [59]. It is overexpressed in squamous cell carcinomas of the cervix. Its knockdown was reported to inhibit the phosphorylation of paired-box gene 8 (PAX8) and the retinoblastoma protein (pRB), as well as to suppress cell cycle-stimulating factors E2F1 and cyclin A2, leading to a decrease in SiHa and HeLa cell proliferation [60]. Stabilizing MSK2 by AAE6 could complement the well-known action of E7 in causing the release of E2F1 through binding to pRB [61].

We previously identified increased signaling in the ERK1/2 pathway for AAE6 compared to EPE6, suggesting that MSK2 is a key contributing factor to increased hypoxia-inducible factor (HIF)-1α seen in AAE6 over EPE6 cells in the hypoxic tumour environment [41]. Interestingly, MSK2 has been shown to activate NF-κB/p65 under different stimuli [62]. It is also known that under hypoxic conditions, NF-κB/p65 is a regulator of HIF-1α gene expression [63]. Although it has not been shown that MSK2 activates NF-κB/p65 under hypoxic conditions, one could speculate that ERK1/2 activation of MSK2 promotes NF-κB/p65 positive regulation of HIF-1α. Consistent with this notion, MSK2 was only targeted by AAE6. Others have likewise observed that ERK1/2 phosphorylation of MSK2 is higher in AAE6 compared to EPE6 and E-T350G (also called L83V) [44]. We conclude that the AAE6 PPI with this kinase may be one of the underlying factors causing the differential finding in sugar metabolism between AAE6- and EPE6-transduced PHFKs previously reported by us [39,41]. Notably, Reactome did not yield pathways related to hypoxia with MSK2 (Table S5).

PROK2 along with PROK1 are chemokine-like proteins (attracting leukocytes to an inflammatory site) usually expressed by components of the innate immune system, such as macrophages with specific roles in host defence and angiogenesis during virus-related cancers [64]. They are ligands transducing their signals through G protein-coupled receptors [65]. PROK2 expression is upregulated in cases of human Merkel cell carcinoma (MCC) containing the MC polyomavirus (MCpyV) [64]. MCpyV shares similarity with HPV via the large T antigen and E6, respectively, targeting P53. This, and the augmentation of tumour-infiltrating macrophages, resulted in positive survival outcomes while the opposite result was obtained with increased PROK1 expression and absence of MCpyV. Using a colorectal cancer cell and mouse model, PROK2 promoted angiogenesis, leading to an increase in colon tumour mass [66]. Interestingly, PROK2 is known to sequester the promoter of HIF-1, alluding to the hypoxic tumour environment (resonating with MSK2 activities above) and to alter the extracellular matrix, potentially controlling angiogenesis [67,68]. PROK2 was found in seven Reactome pathways mostly related to receptor-mediated signal transduction but not to hypoxia, each associated with AAE6 and EPE6, albeit with different entities (Table S5). Taken together, PROK2 may be another interesting AAE6 target, as its dual role in HIF-1 signaling and immune biology may contribute to the “success” of this sub-lineage in tumour development.

#### 2.1.2. EPE6 Interferes with Host-Cellular Immune Surveillance

Mx GTPases MX1 and MX2 are “dynamin-like antiviral machines of innate immunity” [69]. They are essential components of the antiviral response induced by type I and III interferon (IFN) and act as inhibitors of early viral replication [69]. Two MX2 isoforms (with or without nuclear localisation signal) are found in the nucleus or cytoplasm where they interact with different key viral components. For example, during HIV infection, MX2 targets viral genome uncoating, nuclear uptake, and integrase activity of the pre-integration complex. MX2 also has a potential function in cervical carcinogenesis. In the HPV16-positive cell line W12, the selection of infected keratinocytes with integrated viral DNA requires the loss of the episomal genome and that episomal expression of E2 could limit transcription of the integrated viral DNA [70]. Microarray analysis revealed that episome loss was associated with the expression of type I IFN pathway-inducible genes, including MX1 and 2 [70]. Interaction of EPE6 with MX2 and the subsequent potential alteration of MX2 activity could therefore be a factor explaining the different genome integration capacity of AAE6. Based on Y2H experiments, MX2 interacts with the histone-lysine N-methyltransferase EHMT2 [71], which increases P53-dependent expression of pro-apoptotic genes, i.e., Bax and PUMA [72]. EHMT2 is also a known interactor of EP300/CBP [72], which in turn is a known E6 binder (above and Table S1). Reactome pathways for both AAE6 and EPE6 were related to IFN signaling and antiviral mechanisms but with different interactors (Table S5).

#### 2.1.3. AAE6 and EPE6 Both Disrupt P53 Activities

NOG2—a GTPase—is involved in the regulation of G1 to S phase transition [73] and ribosomal biogenesis [74]. NOG2 induces cell proliferation by increasing TP53 (and its downstream product, CDKN1A/p21) protein levels, and decreasing RPL23A protein levels [73]. Interest in NOG2 arose from the observation that the protein is overexpressed in some cancers, such as breast or colorectal [75]. NOG2 induces P53 activation by inhibiting RPL23A (60S ribosomal protein L23a), a ribosomal protein that triggers p53 degradation via Mdm2. P53 expression leads to the expression of the cyclin-dependent kinase inhibitor p21 [73], which is required for the formation of the cyclin D1-CDK4 complex. At higher concentrations, p21 inhibits the cyclin D1-CDK4 complex [76]. An activated cyclin D1-CDK4 complex leads to a decrease in pRB phosphorylation that, in turn, releases E2F1 to promote cell proliferation [73]. NOG2 is a known interactor of IN80B (Supplementary Materials data of [77]) and both proteins interact with RPL23A [73,77]. In addition, NOG2 interacts with MYC [53], which is involved in P53 stabilization. Taken together, these data suggest that NOG2 is involved in several pathways that could all affect P53 activity in the HPV context. An interactome study indicated that in addition to IN80B, NOG2 also interacts with two other proteins selected by the Peptide Method, i.e., ribosome biogenesis protein NSA2 homolog (Nop seven-associated 2, NSA2) [78] and G patch domain-containing protein 4 (GPTC4) [79] (Table S4). These data originate from large interactome studies and the consequences of each interaction were not investigated [78,79]. GPTC4 functions are largely unknown; nevertheless, information available on NSA2 indicates that the protein could be involved in the same pathways as NOG2 as they appeared to have similar functions. No Reactome pathways could be aligned with NOG2 (Table S5).

TRIPC is an E3 ubiquitin-protein ligase that shares similarities with UBE3A (E6AP). It contains the conserved homologues to the E6AP carboxy terminus (HECT) domain and multiple LxxLL (where x denotes any amino acid) motifs that correspond to the E6 binding site on E6AP [7,80,81]. There are four motifs starting at various residues: LQALL (402), LITLL (485), LHFLL (697), and LDQLL (1862) present throughout TRIPC that could potentially allow interaction with E6. TRIPC triggers the ubiquitination and degradation of several proteins, including the P53 activator protein ADP-ribosylation factor (ARF, p14 in humans) [82,83], potentially doubling the effect of P53 inactivation by E6 as ARF acts upstream of E6AP-mediated P53 degradation. Interestingly, TRIPC-dependent ARF degradation is inactivated by MYC or tumour necrosis factor receptor type 1-associated DEATH domain protein (TRADD) binding to TRIPC [84,85]. Since MYC is a known E6 target, E6 binding to TRIPC could promote ARF degradation by stimulating TRIPC activity and E6 binding to TRIPC and/or MYC could protect TRIPC from MYC inactivation. In addition to ARF, the Brahma-related gene 1-associated factor 57 (BAF57) is another protein targeted by TRIPC. BAF57 degradation by TRIPC could be inhibited when BAF155 is bound to TRIPC [86]. BAF57 is a canonical component alongside BAF53 of the SWItch/sucrose non-fermentable (SWI/SNF) chromatin remodeling complex [87], first detected in yeast [88]. Interestingly, BAF53 is essential for the expression of E6 and E7 when the viral genome has been integrated into the host cell [89]. TRIPC is involved in Reactome pathways mainly associated with antigen processing, ubiquitination, and the immune system (Table S5). Interestingly, E6AP is found in all the pathways involving TRIPC and all pathways involving TRIPC are connected to AAE6 and EPE6 but with different entities (Table S5).

CHM4B is thought to be a subunit of the endosomal sorting complex required for transport (ESCRT)-III complex involved in the formation of multi-vesicular bodies and is important in membrane fission processes, including the budding of enveloped viruses [90,91]. CHM4B is overexpressed in hepatocellular carcinomas [91] and some head and neck squamous cell carcinomas [92]. Co-IP experiments indicated that CHM4B interacts with the inhibitory P53 isoform Δ133p53α that can block the activity of full-length P53 [93]. Other interesting interactors of CHM4B are interferon-responsive factor (IRF)-2 [94], breast cancer-associated protein (BRCA) 2 [95], and E-cadherin [96]. CHM4B’s presence in Reactome is mostly associated with antigen processing, HIF viral life cycle, and autophagy, some of which are shared among both variants while others differ (Table S5), e.g., late endosomal microautophagy pathways also include hemoglobin subunit beta HBB (P68871) (AAE6 and EPE6), as well as HIV infection pathways, which also include E3 ubiquitin-protein ligase RBX1 (P62877), tyrosine-protein kinase HCK (P08631) (AAE6), cyclin-dependent kinase 9 CDK9 (P50750), and F-box/WD repeat-containing protein 1A FBW1A (Q9Y297) (EPE6).

### 2.2. Protein-Pathway Method: AAE6 Is More “Successful” than EPE6 in the Malignant Transformation Process

We also designed the more inclusive Protein-Pathway Method to select additional potential E6 targets. We reasoned that, although it is possible that LC-MS/MS will miss several proteins in a given sample, it is far less likely that a whole pathway will be missed. Emphasis was on differences in proteins between two groups rather than on peptide abundance alone, i.e., AAE6 versus PHFK-HA, EPE6 versus PHFK-HA, and AAE6 versus EPE6. We used the same 878 protein candidates as described under the Peptide Method (Section 2.1, Table S3). Each group of proteins was analysed using Reactome to identify the pathway involving these proteins: 251/498 AAE6-targeted proteins were matched to 861 pathways, 199/380 EPE6-targeted proteins to 794 pathways, and 323/586 “unspecific” protein binders in PHFK-HA were matched to 954 pathways. We then compared the pathways obtained for each group and selected those that aligned with AAE6 and/or EPE6 interactors but not with PHFK-HA interactors. We reasoned that pathways only targeted by AAE6 or EPE6 would be most specific, leaving us with 90 unique proteins for AAE6, 61 unique proteins for EPE6, and 20 additional proteins common to both, i.e., altogether 171 potential E6 binders (Table S6). These proteins were then run independently (all AAE6 interactors and all EPE6 interactors separately) on Reactome to assess which pathways were significantly over-represented for each E6 sub-lineage. Within the 25 most significant pathways, 26/90 entities were identified for AAE6 (Table S7A) and 48/61 for EPE6 (Table S7B). Out of 20 overlapping AAE6/EPE6 targets, 5 were identified within the 25 most significant pathways targeted by both variants, whereas 8 were only seen in EPE6-targeted pathways (Table S7C). While the *P*-value was significant throughout for both sub-lineages and the overlapping proteins, the FDR was statistically significant (*P* < 0.05) in 18/25 pathways for AAE6 only (Table S7A–C). Nevertheless, for comparison, we investigated results of both E6 sub-lineages and overlapping E6 targets. AAE6 and EPE6 entities belonging to these pathways are also listed.

#### 2.2.1. AAE6 Affects Notch Signaling, Hypoxia, Metabolism, and DNA Base Excision Repair

AAE6 was strongly associated with 11 Notch1 signaling pathways (**1,2,3,9,10,11,12,13,15,16**,**22**) with the same protein targets (Table S7A): cyclin-dependent kinase 8 (CDK8, P49336), histone acetyltransferase KAT2A (Q92830), E3 ubiquitin-protein ligase RBX1 (P62877), and two isoforms of the F-box/WD repeat-containing protein 7 (FBXW7, Q969H0-1, Q969H0-4). CDK8 phosphorylates the neurogenic locus notch homolog protein 1 (NOTC1), targeting it for ubiquitination and degradation through the proteasome. Consequently, NOTC1 is not acetylated by mastermind-like protein 1 (MAML1) and its transcription is not enhanced by EP300 [97]. Notch signaling regulates cell homeostasis (balancing proliferation, differentiation, and survival/apoptosis), while aberrant Notch signaling seems to be a major factor in driving epithelial neoplasia. Indeed, it was recently reported that “rare driver mutations in head and neck squamous cell carcinomas converge on Notch signaling” [98]. RBX1 and FBXW7 are also part of Wnt signaling (pathway **14**), suggesting communication between these two pathways. Wnt/beta catenin signaling plays a role in development and adult homeostasis. In the latter, it is mostly inactive and controlled by kinases, such as glycogen synthase kinase 3 (GSK3), casein kinase 1 (CK1), axin, and adenomatous polyposis coli (APC), a tumour suppressor gene often mutated in colon cancer ([99] and references therein). Interestingly, using our Peptide Method, APC (P25054) was found only once with just one unique peptide, yet it appeared in pathway **14** targeted by AAE6.

We also found AAE6 binders belonging to defective base excision repair (BER) associated with the human homologue of the *Escherichia coli* mutY gene (hMYH), i.e., two isoforms of its encoded protein adenine DNA glycosylase (MUTYH): Q9UIF7-3 and -6 (pathways **5,7**,19). MUTYH germline mutations of BER (pathways **5,7**,19) cause MUTYH-associated polyposis (MAP), a disorder similar to familial adenomatous polyposis (FAP), caused by mutations in the APC gene [100]. This association between AAE6 and BER indicates that the AA (D2/D3) sub-lineage integrates earlier into the host genome than EP (A1), supported by observations in an organotypic cell culture model of early cervical carcinogenesis [40,101]. Indeed, another group reported that BER is essential for the HIV provirus DNA to integrate into the host genome, proposing an interesting analogy with transposable elements [102]. In “the landscape of viral association with human cancers” study led by the Pancancer Analysis of Whole Genomes Consortium [103], HPV16 integration seems to be associated with fragile sites or regions with limited access to DNA repair complexes. Our findings from the peptide and protein method corroborate these results: ATP-dependent chromosome remodeling complex INO80 implicated in DNA repair enhances BER activity [104], and the antiviral function of MX2 resulting in viral integration [70] would “help” AAE6 but not EPE6 to insert itself into the host cell genome.

We observed AAE6 binding to the following P53-regulated metabolic genes (pathway **8**): P53 (P04637), MTOR (P42345), thioredoxin (THIO, P10599), trinucleotide repeat-containing gene 6C protein (TNR6C, Q9HCJ0), cytochrome c oxidase subunit 6B1 (CX6B1, P14854), and 5’-AMP-activated protein kinase subunit gamma-2 (AAKG2, Q9UGJ0). Note that P53-regulated metabolic genes also communicate with Wnt signaling via AAKG2. Hence, AAE6-targeted cellular proteins derive from the axis of Wnt and Notch1 signaling, as well as Wnt signaling and P53-regulated metabolic genes. The most striking candidates for this axis are RBX1, FBXW7, KAT2A, and CDK8, due to their presence in 8 to 11 pathways (Table S7A).

AAE6 targeting of pathway **8** proteins (and its association with MSK2, as discovered in the Peptide Method) explains how this variant deregulates cellular metabolism via the Warburg effect [39,41] and promotes a hypoxic environment via elevated HIF-1α levels [41]. Deregulated energetics and hypoxia—two hallmarks of cancer [105]—may cause the “higher” carcinogenic ability of AAE6. While EPE6 also targets P53, CX6B1, and THIO of pathway **8**, AAE6 may have an advantage over EPE6 due to the additional actions of MTOR, TNR6C, and AAKG2. Moreover, RBX1, FBXW7, and CDK8 (Notch1 signaling pathways **1,2,3,9,10,11,12,13,15,16**), as well as MTOR (pathway **8**), are known HIF-1α binders [106,107,108], reinforcing AAE6′s effect on hypoxia. Indeed, alteration of MTOR function is known to promote HIF-1α translation [109]. CDK8 and its associated protein mediator of RNA polymerase II transcription subunit 1 and 26 are recruited by the HIF-1α transactivation domain at the promoter of several hypoxia-inducible genes, leading to the activation of the super elongation complex (SEC) to promote RNA polymerase II (RNAPII) elongation [106]. In addition, proto-oncogenes c-Fos-encoded protein (FOS, P01100) (pathways 21, 24) and 6-phosphofructo-2-kinase/fructose-2,6-bisphosphatase 3 (F263, Q16875) were found to be associated with HIF-1α, despite not directly interacting with it [110,111]. HIF-1α and FOS both bind the vascular endothelial growth factor promoter under hypoxic conditions [110]. F263, a master regulator of glycolysis, is activated by HIF-1α and could be of importance for Warburg effect development [112].

Finally, Forkhead box protein O1 (FOXO)-mediated transcription (pathway 25 with AAE6 interactors and pathway 5 with proteins common to AAE6 and EPE6) overlaps the AAE6 and EPE6 variants via nuclear transcription factor Y subunit alpha NFYA (P23511) and THIO (Table S7C). AAE6 also interacts with the ATP-binding cassette sub-family A member 6 (ABCA6, Q8N139) and F-box only protein 32 (FBX32, Q969P5), which are involved in the FOXO-mediated transcription pathway, indicating that AAE6 could disrupt the pathway more efficiently than EPE6. Of interest, FOXO1 expression is decreased in CaSki and SiHa cells, but the regulation of FOXO1 in cervical cancer is not yet fully understood [113]. FOXO transcription factors act in pathways controlling cell survival, growth, differentiation, and metabolism in various scenarios, such as growth factor deprivation, starvation, and oxidative stress [114].

#### 2.2.2. EPE6 Is Associated with DNA Damage Repair and Cancer Pathways

Up to eight EPE6 interactors are part of homology-directed repair (HDR) DNA damage pathways 5,9,19,22: P53, THIO, proliferating cell nuclear antigen (PCNA, P12004), CX6B1, double-strand break repair protein MRE11 (P49959), cyclin-dependent kinase 9 (CDK9, P50750), geranylgeranyl transferase type-2 subunit alpha (PGTA, Q92696), and DNA endonuclease RBBP8 (Q99708). PCNA also interacts with the adenine DNA glycosylase MUTYH [115], linking the two E6 variants. However, while some of the targeted pathways overlap, most of the entities associated with the individual E6 variant differ (Table S5). Like AAE6, EPE6 seems to target various pathways crucial for carcinogenesis, such as Hippo, PI3K/AKT, growth factor-initiated mesenchymal-epithelial signal transduction (MET), and epithelial growth factor receptor (EGFR) (pathways 1,3,7,10,14,24). Hippo communicates with the Wnt pathway through segment polarity protein dishevelled homologue DVL-2 (DVL2, O14641) via EPE6′s pathway 1 and AAE6′s pathways **14** and **17**. The most notable EPE6 target seems to be PI3K regulatory subunit alpha (P85A, P27986), detected in five pathways related to PI3K, MET, and EGFR (3,7,10,14,24), which all communicate with each other (Table S7B). Interestingly, some HIF-1α binders were also found to be targeted with EPE6, i.e., WW domain-containing transcription regulator protein 1 (WWTR1, Q9GZV5) (pathway 1) and CDK9 (pathway 22) [106,116]. In breast cancer, WWTR1 (also called transcriptional co-activator with PDZ-binding motif) expression is activated by HIF-1α and is important for the maintenance of breast cancer stem cells [117]. Since CDK9 is a component of SEC recruited by CDK8 upon interaction with HIF-1α [106], both AAE6 and EPE6 could potentially alter RNAPII elongation. Nevertheless, only CDK8 is essential to the process [106], suggesting that AAE6 and EPE6 work differently to modify RNAPII elongation.

#### 2.2.3. AAE6 and EPE6 Both Prevent P53 Activity

The 25 most significant pathways generated for the 20 overlapping interactors of AAE6 and EPE6 are listed in Table S7C. No differential pathway enrichment was noted, as all 124 pathways showed the same FDR value of 0.09. Out of 20 overlapping proteins, 15 were distributed among the top 25 pathways, with 13 found in the pathways described for AAE6 and EPE6: P53, NFYA, DVL2, THIO, CX6B1, CHM4B, HBB, inactive polyglycylase tubulin tyrosine ligase-like protein 10 (TTL10, Q6ZVT0), tubulin monoglycylase TTLL3 (Q9Y4R7), SH3 domain-containing kinase-binding protein 1 (SH3K1, Q96B97), fibrinogen alpha chain (FIBA, P02671), coiled-coil domain-containing protein 59 (TAP26, Q9P031), and testisin PRSS21 (Q9Y6M0). Consequently, most pathways identified with these proteins were involved in the same pathways as those described for AAE6 or EPE6 above, albeit with fewer entities (Table S7C). These results suggest that several pathways are targeted by both AAE6 and EPE6 but with different efficiencies (Table S7C). The two remaining proteins, protein lin-37 homologue (Q96GY3) and centromere protein R (Q13352), were found in pathway 14 (Cell cycle, Mitotic) alongside P53 and CHM4B. Overall, P53 is the main contributor found in 13 of the 25 pathways (1,4,7,10,12,13,14,18,19,20,23,24; Table S7C), and is the only entity found in 9 of them. Thus, P53 appears to be the most important target between AAE6 and EPE6.

## 3. Discussion

**Figure 3:**
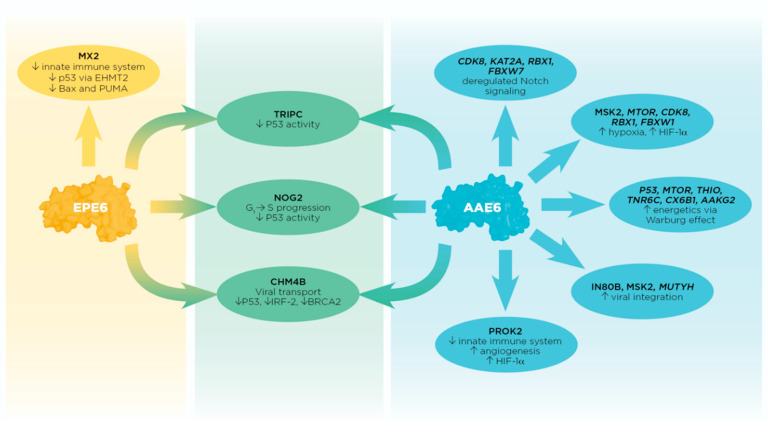
Summary of individual and tandem effects between AAE6 and EPE6 PPIs with host cellular proteins by which E6 may promote cell-transforming processes. Candidates from both approaches are shown for AAE6 only due to the lack of significant FDR values for EPE6 and AAE6/EPE6 combined using the Protein-Pathway Method (upright font = Peptide Method, italic font = Protein-Pathway Method). Candidates from the Peptide Method are shown for AAE6, EPE6, and AAE6/EPE6 (upright font). The first-described E6 binder (P53; Table S1) is most likely targeted through several mechanisms shared by both E6 variants (**middle**, **in green**). AAE6 and EPE6 proteins equally bound 3 proteins known to affect P53 inactivation: TRIPC, CHM4B, and NOG2. Collectively, these interactions could result in decreased P53 function far beyond its well-described E6- and E6AP-mediated degradation through the proteasome. Potential interactions unique to AAE6 (**right**, **in blue**) are mostly associated with Notch signaling, hypoxia, energetics, DNA base excision repair, and to some extent with the innate immune system. Notably, some AAE6-targeted molecules have multiple roles, e.g., MTOR is associated with hypoxia and metabolism, CDK8 with hypoxia and Notch signaling, and MSK2 with hypoxia and chromatin remodeling, further underlining this variant’s “lead” in the malignant process. AAE6′s association with MUTYH, IN80B, and MSK2 could promote its integration potential and the binding to PROK2 could promote both angiogenesis and hypoxia within infected cells. Being a chemokine-like molecule, PROK2 is implicated in the innate immune system normally attracting macrophages to the site of inflammation. In the hypoxic and consequently more acidic tumour environment, tumour-associated macrophages (TAMs) may develop from original site-filtrating macrophages adapting to the tumour microenvironment. Tumour growth is then promoted by the positive feedback loop between TAMs and epithelial cells via the expression of colony-stimulating factor and epithelial growth factor, respectively. EPE6′s binding to MX2 (**left**, **in yellow**) may limit this variant’s ability to integrate into the host genome effectively while it may be more successful in deregulating the host’s viral defence. MX2 also affects the P53 pathway, e.g., via EHMT2 and the expression of pro-apoptotic genes Bax and PUMA, further duplicating well-established anti-P53 E6 activities.

This study is one of few reporting PPIs in a global perspective focusing on two common E6 sub-lineages with varying prevalence in cervical cancer. Using two different approaches to dissect the AAE6 and EPE6 variants and their cellular interactors (Figure 3), it was clear that AAE6 takes the lead in targeting hypoxia and energetics, while both E6 proteins equally inactivate p53 by multiple means. The latter observation, along with the fact that AAE6 is also very active in subverting Notch1 signaling, are completely new discoveries and are strengthened by the finding that hypoxia requires Notch1 signaling to maintain the undifferentiated cell state in various stem and precursor cell populations [118]. The identification of the viral defence protein MX2 targeted by EPE6, making this variant less likely to integrate into the host cell genome than AAE6, bolsters our earlier in vitro data describing this phenomenon [40,101]. Targeting BER by AAE6 further underlines these findings. Subtle and indirect changes in viral immune evasion mechanisms were also uncovered for both variants. We are mindful that our results must be validated with additional wet lab experiments, such as reverse co-IP and RNA interference, to elucidate gain or loss of function of reported candidate binders. Although only a small snapshot of identified proteins was provided here, confirming these new discoveries will provide vital information about E6′s ability to drive tumourigenic processes. Our study strengthens the notion that E6 is an excellent target for an anti-HPV treatment [47], whose design would need to consider E6 sub-lineage differences.

## 4. Materials and Methods

### 4.1. Wet Lab Methods

Cell culture, cell lysis, Western blot, and co-immunoprecipitation (co-IP) methods used in this investigation are described in detail in the Supplementary Materials data. The most challenging step in optimizing co-IP was protein elution, which was limited by several factors: compatibility in downstream liquid chromatography tandem mass spectrometry (LC-MS/MS) applications, the effectiveness of removing target proteins, and minimizing antibody leeching. Elution methods described by other groups [48,119] did not elute detectable levels of 16E6 in every replicate. Acidic elution was chosen for this study, since it was most effective at elution and also compatible with LC-MS/MS.

### 4.2. Liquid Chromatography Tandem Mass Spectrometry (LC-MS/MS)

Two independent mass spectrometry trials were conducted with the two hemagglutinin-tagged (HA) variants: AAE6-HA, EPE6-HA, and HA vehicle-transduced primary human foreskin keratinocytes (PHFK-HA) [38] treated with the proteasome inhibitor MG132-DMSO or DMSO (both from Millipore Sigma, Burlington, MA, USA) only, to yield the highest possible number of E6-binding partners [48]. Following co-IP and elution, all samples were shipped to the Harvard Center for Mass Spectrometry (HCMS), Cambridge, MA, USA and processed as a paid service on demand. Detailed information can be found in the Supplementary Materials data.

### 4.3. Databases Used for Contaminant Removal and Identification of Cellular Targets

To determine the relationships between proteins identified by LC-MS/MS, we used three regularly curated and freely accessible databases: the Universal Protein Resource (UniProt) [120] for molecular functions and biological processes; the Biological General Repository for Interaction Datasets (BioGRID) [121] for PPI analysis; and the Reactome Pathway Database (Reactome) [122] to map E6 protein binders to the biological pathway in which they are involved. UniProt (whose protein abbreviations we adopted and reported in our study) provides an accession number (shown in our results) for each protein, which can be inserted into Reactome or BioGRID. Reactome generates a ranking of the 25 most significant pathways, meaning that these pathways are over-represented compared to others based on their probability value (*P*-value) and false discovery rate (FDR) [123]. In our context, Reactome was used to discriminate non-specific binders and to determine how cellular pathways are (differentially) targeted by AAE6 vs. EPE6 and BioGRID if proteins of interest were interacting with each other.

### 4.4. Post LC-MS/MS Data Contaminant Removal

We merged the original “raw” HCMS Excel (Microsoft, Redmond, Washington, United States) files that listed each trial (T1 and T2), condition (DMSO and MG132), E6 variant (AAE6 or EPE6), and PHFK-HA. After manual removal of sample-processing contaminants labelled *CON* (*n* = 50) from the merged Excel spreadsheet, we obtained a raw heatmap with a total of 1584 proteins listed in alphabetical order (Table S2). We then cleaned the data of ribosomal proteins (*n* = 95) by running them through a Reactome [123] analysis using all the remaining proteins, then further removed proteins involved in pathways of RNA metabolism (*n* = 89), yielding 1464 remaining proteins (Table S3). Proteins present in any of the PHFK-HA samples were likely to be contaminants and were likewise removed from the Excel spreadsheet (*n* = 586; Table S3). After contaminant removal, 878 proteins remained (498 proteins in AAE6 and 380 in EPE6) (Table S3); of these, *n* = 108 overlapped.

## Supplementary Materials

Supplementary materials can be found at https://www.mdpi.com/1422-0067/21/21/7980/s1.
