# Supplementary material for: Virus–Host Protein–Protein Interactions between Human Papillomavirus 16 E6 A1 and D2/D3 Sub-Lineages: Variances and Similarities
Source: Int J Mol Sci. 2020 Oct 27;21(21):7980. doi: 10.3390/ijms21217980 (PMC7663357; doi:10.3390/ijms21217980)
Supplement: Supplementary file 1 [file ijms-21-07980-s001.zip › ijms-966769-supplementary/ijms-967769 suppl/reference-supplementary data.docx]

**References (Supplementary data)**

1. Free, R.B.; Hazelwood, L.A.; Sibley, D.R. Identifying novel protein-protein interactions using co-immunoprecipitation and mass spectroscopy. *Curr. Protoc. Neurosci.* **2009**, *Chapter 5*, Unit-5.28, doi:10.1002/0471142301.ns0528s46.
2. Werness, B.A.; Levine, A.J.; Howley, P.M. Association of human papillomavirus types 16 and 18 E6 proteins with p53. *Science* **1990**, *248*, 76–79, doi:10.1126/science.2157286.
3. Oliveira, L.B.; Haga, I.R.; Villa, L.L. Human papillomavirus (HPV) 16 E6 oncoprotein targets the Toll-like receptor pathway. *J. Gen. Virol.* **2018**, *99*, 667–675, doi:10.1099/jgv.0.001057.
4. Nakagawa, S.; Huibregtse, J.M. Human Scribble (Vartul) Is Targeted for Ubiquitin-Mediated Degradation by the High-Risk Papillomavirus E6 Proteins and the E6AP Ubiquitin-Protein Ligase. *Mol. Cell. Biol.* **2000**, *20*, 8244–8253, doi:10.1128/mcb.20.21.8244-8253.2000.
5. Jeong, K.W.; Kim, H.Z.; Kim, S.; Kim, Y.S.; Choe, J. Human papillomavirus type 16 E6 protein interacts with cystic fibrosis transmembrane regulator-associated ligand and promotes E6-associated protein-mediated ubiquitination and proteasomal degradation. *Oncogene* **2007**, *26*, 487–499, doi:10.1038/sj.onc.1209837.
6. Jing, M.; Bohl, J.; Brimer, N.; Kinter, M.; Vande Pol, S.B. Degradation of Tyrosine Phosphatase PTPN3 (PTPH1) by Association with Oncogenic HumanPapillomavirus E6 Proteins. *J. Virol.* **2007**, *81*, 2231–2239, doi:10.1128/jvi.01979-06.
7. Vos, R.M.; Altreuter, J.; White, E.A.; Howley, P.M. The Ubiquitin-Specific Peptidase USP15 Regulates Human Papillomavirus Type 16 E6 Protein Stability. *J. Virol.* **2009**, *83*, 8885–8892, doi:10.1128/jvi.00605-09.
8. Nusse, R.; Brown, A.; Papkoff, J.; Scambler, P.; Shackleford, G.; McMahon, A.; Moon, R.; Varmus, H. A new nomenclature for int-1 and related genes: The Wnt gene family. *Cell* **1991**, *64*, 231, doi:10.1016/0092-8674(91)90633-A.
9. Artavanis-Tsakonas, S.; Rand, M.D.; Lake, R.J. Notch Signaling: Cell Fate Control and Signal Integration in Development. *Science* **1999**, *284*, 770, doi:10.1126/science.284.5415.770.
