# Supplementary material for: Virus–Host Protein–Protein Interactions between Human Papillomavirus 16 E6 A1 and D2/D3 Sub-Lineages: Variances and Similarities
Source: Int J Mol Sci. 2020 Oct 27;21(21):7980. doi: 10.3390/ijms21217980 (PMC7663357; doi:10.3390/ijms21217980)
Supplement: Supplementary file 1 [file ijms-21-07980-s001.zip › ijms-966769-supplementary/ijms-967769 suppl/Table S1.pdf]

Supplemental Table 1 – Cellular targets of the human papillomavirus type 16 E6 protein. The first study encountered in our survey was that by Werness et al. (1990) [125] and the last one that by Oliveira et al. (2018) [126]. Most commonly used methods for PPIs were the yeast two hybrid (Y2H) screen, glutathione S-transferase (GST) pulldown, immunoprecipitation (IP) and co-IP. Six papers also reported some form of mass spectrometry (MS) [18,23,48,128-130]. There is some confusion about protein nomenclature: in the first column (left), we refer to an alias if the UniProt protein nomenclature (The UniProt Consortium 2019) [120] differs from that used in the publication, e.g. in the title and/or abstract; in the other columns, we use the nomenclature adopted by the respective publication. When looking at the binding mechanism, we considered the domain of the targeted protein regarding its amino acid sequence, region (N- or C-terminus) where this information was available. Using the UniProt database (The UniProt Consortium 2019) [120], we have collectively interpreted the data of all 50 binders together in the light of biological functions. Viral processes and immune response, mitogen-activated protein kinases (MAPK)/extracellular signal-regulated kinases (ERK) cascade as well as Wnt (portmanteau of Wingless and int-1, [131]) and Notch ("notches at the Drosophila wing margin", [132]) signaling pathways—both highly conserved in evolution and embryogenesis-related, were mostly targeted followed by tumour suppressor proteins, DNA damage and repair, apoptosis, adhesion, immortalization and transformation, cell cycle and proliferation, and transcription. In line with these observations, a recent publication dissecting viral associations in patient specimen of the Pan-Cancer Analysis of Whole Genomes Consortium, impaired antiviral defence mechanisms were found to be the driving force for HPV16-related malignancies including cervical, bladder and head and neck cancers [103].

**Protein Name**	**Accession Number**	**Binding Mechanism**	**Method**	**Outcome of Interaction**	**Reference**
(1) Transcriptional adapter 3 (TADA3) alias hADA3	O75528	Targets hADA3 to prevent co-activation of p53	Y2H and GST pulldown	Preventing p53-mediated transactivation of target promoters and p53 stabilization	Kumar et al. 2002
(2) Bcl-2 homologous antagonist/killer (BAK1)	Q16611	Targets BAK1 through E6AP known to interact with it	GST pulldown	Part of the pro-apoptotic Bcl-2 family; degraded by E6 thereby preventing BAK-induced apoptosis	Thomas and Banks 1999
(3) BRCA1-associated RING domain protein 1 (BARD1)	Q99728	BARD1 binds to E6 through E6's two zinc finger motifs with zinc finger 1 (AA 30-66) being the most important region for binding	Y2H and IP	Tumour suppressor function through apoptotic signaling inhibited	Yim et al. 2007

Protein Name	Accession Number	Binding Mechanism	Method	Outcome of Interaction	Reference
(4) Breast cancer type 1 susceptibility protein (BRCA1)	P38398	Targeted via zinc finger domains of E6	GST pulldown	Inhibitory telomerase activity inactivated by E6	Zhang et al. 2005
(5) Golgi-associated PDZ and coiled-coil motif-containing protein (GOPC) alias CAL	Q9HD26	The PDZ domain of CAL interacts with the PDZ-binding motif of E6; interacts with the E6/E6AP complex enhancing proteasome degradation	GST pulldown and MALDi-TOF	E6 mediates proteasome degradation of CAL through E6AP	Jeong et al. 2007
(6) Histone-arginine methyltransferase CARM (CARM1)	Q86X55	Not acquired	*In vitro* methyltransferase assay	Prevention of p53-responsive promotors and downregulation of p53 downstream gene expression	Hsu et al. 2012
(7) CREB-binding Protein (CREBBP) alias CBP	Q92793	E6 binds to 3 regions on CBP and p300: C/H1, C/H3 and C-terminus; binding is independent of p53	Co-IP	E6 inhibits the activation of p53 and NF-kB by CBP/p300	Patel et al. 1999
(8) Ubiquitin carboxyl-terminal hydrolase CYLD (CYLD)	Q9NQC7	Not acquired	EMSA and NF-kB reporter gene assay	E6 mediated ubiquitination and proteasomal degradation of CYLD resulting in hypoxia-induced NF-kB activation	An et al. 2008
(9) Discs large homologue 1 (DLG1)	Q12959	E6 binds to second PDZ domain via C-terminal XS/TXV/L motif	MBP and GST pulldown	E6 binding to hDLG promotes the transformation of cells	Kiyono et al. 1997
(10) Discs large homologue 4 (DLG4)	P78352	Binds to E6's C-terminus through its second PDZ motif; last amino acid change from leucin to valine changes affinity	MBP and GST pulldown	Suggested tumour suppressor function; E6 binds induces proteolytic degradation of DLG4	Handa et al. 2007
(11) Ubiquitin-protein ligase E3A (UBE3A) alias E6AP	Q05086	Not acquired	Co-IP and GST pulldown	E6 forms a stable complex with E6AP and mediates numerous downstream interactions such as proteolytic degradation of p53	Huibregtse et al. 1991

Protein Name	Accession Number	Binding Mechanism	Method	Outcome of Interaction	Reference
(12) Reticulocalbin-2 (RCN2) alias E6BP, ERC55	Q14257	E6BP residues 18-29 (VSLEEFLGDY) are the binding site for E6	Y2H and GST pulldown	Interaction with E6 forms a complex and is thought to be involved with E6 induced transformation and degradation of cellular proteins	Chen et al. 1995, 1998
(13) Signal-induced proliferation-associated 1-like protein 1 (SIPA1L1) alias E6TP1	O43166	Binds to E6TP1's C-terminus at residue 194; PDZ domain within E6TP1 has little effect on binding with E6	Y2H and GST pulldown	Possible tumour suppressor protein; degradation by E6 potentially alters G-associated protein signaling pathways	Gao et al. 1999
(14) FAS-associated death domain protein (FADD)	Q13158	Targets N-terminus at Serine residue 10, 14, 16 and 18 and Glutamic acid residue 19; site-directed mutants enabled localization of E6-binding to N-terminal end of FADD	Mammalian Y2H and GST pulldown	E6 accelerates depredation of FADD preventing transmission of apoptotic signals through FAS pathway	Filippova et al. 2004
(15) Fibulin-1 (FBLN1)	P23142	No consensus binding motif identified	Y2H and GST pulldown	Inhibiting Fibulin-1 allowing for invasion and metastasis	Du et al. 2002
(16) E3 ubiquitin-protein ligase HERC2 (HERC2)	O95714	Interaction with E6 is E6AP dependent	Co-IP and MS-LC/LC	Interacts with E6 through formation of complex with E6AP to result in degradation of HERC2	White et al. 2012
(17) Protein scribble homolog (SCRIB) alias hScrib	Q14160	Interacts with E6AP in the presence of E6, C-terminus of E6 recognizes PDZ domain of hScrib	GST pulldown and MALDI-MS/MS	Ubiquitination results in degradation and reducing integrity of tight junctions	Nakagawa et al. 2000
(18) Telomerase reverse transcriptase (hTERT)	O94807	Proximal promotor/regulatory regions (nt position -251 to -88 and +5 to +40) involved with 60% of E6-induced hTERT activity	Telomerase activity and mRNA protection assay	E6 induces increased hTERT activity resulting in maintenance of telomere length	Veldman et al. 2001
(19) Inhibitor of nuclear factor kappa-B kinase subunit beta (IKKB)	O14920	Not acquired	Co-IP	E6 binding potentially intervenes with NF-kB activation during bacterial or viral infection or DNA damage	Oliveira et al. 2018

Protein Name	Accession Number	Binding Mechanism	Method	Outcome of Interaction	Reference
(20) Inhibitor of nuclear factor kappa-B kinase subunit epsilon (IKKE)	Q14164	E6 binds to a location within the first 160 residues of IKKE	Co-IP	E6 binding potentially intervenes with NF-kB activation during TLR9 signaling	Oliveira et al. 2018
(21) Interleukin-1 receptor-associated kinase-like 2 (IRAK2)	O43187	Not acquired	Co-IP	E6 binding potentially intervenes with NF-kB activation during TLR9 signaling	Oliveira et al. 2018
(22) Interferon regulatory factor 3 (IRF3) alias IRF-3	Q14653	IRF-3 residues 109-149 containing ELLG sequence (as in E6AP)	Y2H and GST pulldown	Transcriptional activator; interacts with E6 inhibiting transactivation of IFN-ß	Ronco et al. 1998
(23) Membrane-associated guanylate kinase, WW and PDZ domain-containing protein 1 (MAGI1) alias MAGI-1	Q96QZ7	E6 PBM interacts with PDZ1 of MAGI-1	GST pulldown	Functions in signal transduction and likely a tumour suppressor protein; E6 targets MAGI-1 for proteasomal degradation	Glaunsinger et al. 2000
(24) (MAGI2) alias MAGI-2	Q86UL8	PDZ1 domain interacts with E6 most likely through the PDM	*In vitro* degradation assay	Functions in signal transduction; E6 targets MAGI-2 for proteasomal degradation	Thomas et al. 2002
(25) (MAGI3) alias MAGI-3	Q5TCQ9	PDZ1 domain interacts with E6 most likely through the PBM	*In vitro* degradation assay	Functions in signal transduction; E6 targets MAGI-3 for proteasomal degradation	Thomas et al. 2002
(26) DNA replication licensing factor MCM7 (MCM7) alias hMCM7	P33993	Deletion analysis found E6's N-terminal residues 1-91 bind to hMCM7 C-terminal residues 572-719	Y2H	Component of replication licensing factors; E6 potentially interferes with its ability to associate with chromatin avoiding G1-phase arrest point	Kukimoto et al. 1998
(27) Methylated-DNA--protein-cysteine methyltransferase (MGMT)	P16455	MGMT interacts with E6AP through L2G box sequence LLGXXXS/T; PDZ domain present shows potential binding with E6	GST pull-down and IP	DNA repair protein that protects against mutations; E6 promotes ubiquitination-dependent degradation	Srivenugopal et al. 2002
(28) Myc proto-oncogene protein (MYC)	P01106	Binds to E6 in an E6AP dependent manner along with E2F1	GST pulldown	Cellular regulatory processes; E6 binding reduces MYC's half-life and accelerates its degradation	Gross-Mesilaty et al. 1998

Protein Name	Accession Number	Binding Mechanism	Method	Outcome of Interaction	Reference
(29) Myeloid differentiation primary response protein (MyD88)	Q99836	Not acquired	Co-IP	E6 binding potentially prevents innate immune receptor signaling	Oliveira et al. 2018
(30) Nuclear factor kappa B subunit 1/2 (NFkB1/2) alias NF-kB	P19838/Q00653	Not acquired	Immortalization and dual luciferase assays	E6 increased NF-kB levels for baseline and TNF-α by 2- to 3-fold	Vandermark et al. 2012
(31a) Transcriptional repressor (NFX1) alias NFX1-91	Q12986-3 (NFX1-isoform 3)	NFX1-91 is destabilized by the E6/E6AP complex at NFX-91's C-terminus	Y2H, co-IP and RT-qPCR	Transcriptional repressor of hTERT promoter; E6/E6AP complex destabilizes NFX1-91 through ubiquitination	Gewin et al. 2004
(31b) Transcriptional repressor (NFX1) alias NFX1-91	Q12986-1 (NFX1-isoform 1)	NFX1-123 is stabilized in the presence of E6	Y2H, co-IP, GST pulldown and LC-MS/MS	Transcriptional activator of hTERT promoter; E6 may bring NFX1-123 to the hTERT promoter allowing for increased hTERT activation and overexpression	Katzenellenbogen et al. 2007
(32) InaD-like protein (PATJ)	Q8NI35	E6 binds to the PDZ domain of PATJ (ETQL)	Y2H and co-IP	E6 binds to and targets PATJ for degradation independently of E6AP preventing the formation of a TJ-associated complex Par6-aPKC-PAR3 responsible for regulating kinase activity and formation of tight junctions in polarized cells	Storrs and Silverstein 2007
(33) Paxillin (PAXI)	P49023	E6 effect likely occurs downstream of paxillin tyrosine phosphorylation and is sensitive to status of actin polymerization	GST pulldown	Transduces signals from plasma membrane to focal adhesions and actin cytoskeleton; E6 disrupts paxillin-mediated actin formation	Tong et al. 1997
(34) E3 ubiquitin-protein ligase PDZRN3 (PDZRN3)	Q9UPQ7	PDZRN3 interacts with E6 within the PBM	Y2H	When interacting with E6, PDZRN3 is targeted for degradation increasing STAT5-ß activation	Thomas and Banks 2015

**Protein Name**	**Accession Number**	**Binding Mechanism**	**Method**	**Outcome of Interaction**	**Reference**
(35) Serine/ threonine-protein kinase N1 (PKN1)	Q16512	E6 binds to C-terminal region of PKN	Y2H and GST pulldown	PKN1 phosphorylates E6, which may allow for E6 to influence Rho-mediated signaling	Gao et al. 2000
(36) Protein arginine N-methyltransferase 1 (PRMT1)	Q99873	Not acquired	*In vitro* methyltransferase assay	E6 reduces PRMT1-induced methylation of histone H4 at R3 resulting in reduced p53 transactivation	Hsu et al. 2012
(37) Caspase-8 (CASP8) alias Procaspase-8	Q14790	Not acquired but both E6 full-length and truncated E6* can bind to procaspase 8	Mammalian Y2H, GST pulldown, IP and co-IP	E6 targets procaspase 8 for degradation decreasing its interaction with FADD and procaspase 8 dimerization; truncated E6 stabilizes procaspase 8	Filippova et al. 2007
(38) Tyrosine-protein phosphatase non-receptor type 3 (PTPN3)	P26045	Interaction between C-terminus of E6 and the PDZ domain of PTPN3	GST pulldown and LC-MS/MS	Membrane-associated phosphatase; degraded by E6 which prevents tyrosine phosphorylation of growth factor receptors	Jing et al. 2007
(39) Cellular tumour antigen p53 (P53)	P04637	Not acquired	IP	E6 binds to and degrades p53	Werness et al. 1990
(40) Histone acetyltransferase p300 (EP300)	Q09472	Binding domain on E6 between residues 100-147; binds to 3 regions on CBP/p300: C/H1, C/H3 and C-terminus	Co-IP	Coactivator important for cell differentiation and cell cycle progression; E6 prevents the activation of p53 and NF-kB via CBP/p300	Patel et al. 1999
(41) Histone-lysine N-methyltransferase SETD7 (SETD7)	Q8WTS6	Not acquired	*In vitro* methyltransferase assay	Methylation of histones and non-histone substrates such as p53; inhibition by E6 results in the decrease of p53 stability and activity	Hsu et al. 2012
(42) Telomerase reverse transcriptase (TERT)	O14746	Not acquired	Modified TRAP	E6 causes ubiquitin-mediated degradation of a telomerase repressing protein	Klingelhutz et al. 1996

Protein Name	Accession Number	Binding Mechanism	Method	Outcome of Interaction	Reference
(43) Tax-1 binding protein 3 (TX1B3) alias TIP-1	O14907	TIP-1 interacts with E6 by binding to the PDZ binding region at E6's terminus	Y2H confirmed with co-IP	TIP-1 interacts with E6 but rather than being degraded, it results in increased activation of RhoA kinase	Hampson et al. 2004
(44) Histone acetyltransferase KAT5 (KAT5) alias TIP60	Q92993	Charged residues of the N-terminus of E6	GST pulldown	E6 destabilizes and degrades TIP60 promoting cell proliferation and cell survival	Jha et al. 2010
(45) Tumor necrosis factor receptor superfamily member 1A (TNR1A)	P19438	E6 binds to the C-terminal cytoplasmic tail of TNF R1	IP and mammalian Y2H	E6 inhibits TNF induced apoptosis and formation of the death induced signaling complex (DISC	Filippova et al. 2002
(46) TIR domain-containing adapter molecule 1 (TCAM1) alias TRIF	Q8IUC6	Not acquired	Co-IP	E6 binding potentially inhibits innate immune functions and antiviral responses	Oliveira et al. 2018
(47) TNF receptor-associated factor 6 (TRAF6)	Q9Y4K3	Not acquired	Co-IP	E6 binding potentially de-regulates DNA damage response and host immunity	Oliveira et al. 2018
(48a) Tuberin (TSC2)	P49815	Residues 1-175 and 1251-1807 of TSC2 are required for binding to residues of 260-316 and 428-500 of E6AP	GST pulldown and co-IP	E6 binds to E6AP/TSC2 complex and targets TSC2 for degradation	Zheng et al. 2008
(48b) Tuberin (TSC2)	P49815	DILG and ELVG domains of Tuberin bind to E6 residues 78-104	Y2H and GST pulldown	E6 binding causes its ubiquitin-mediated degradation	Lu et al. 2004
(49) Ubiquitin carboxyl-terminal hydrolase 15 (UBP15) alias USP15	Q9Y4E8	Not acquired	Targeted MS	Results in increased stability and increased E6 half-life	Vos et al. 2009
(50) DNA repair protein XRCC1 (XRCC1)	P18887	E6 interacts with the N-terminus of XRCC1 (residues 107-170)	Y2H and co-IP	E6 inhibition prevents ability to maintain genetic integrity and utilize DNA strand break repair mechanisms	Iftner et al. 2002
