# Supplementary material for: Virus–Host Protein–Protein Interactions between Human Papillomavirus 16 E6 A1 and D2/D3 Sub-Lineages: Variances and Similarities
Source: Int J Mol Sci. 2020 Oct 27;21(21):7980. doi: 10.3390/ijms21217980 (PMC7663357; doi:10.3390/ijms21217980)
Supplement: Supplementary file 1 [file ijms-21-07980-s001.zip › ijms-966769-supplementary/ijms-967769 suppl/Table S7.pdf]

Table A – **Reactome analysis of the AAE6 interactors selected with the Protein-Pathway method. The pathways with a significant FDR (<0.05) are bolded**

Pathway name	Entities found	Entities p-value	Entities FDR	UniProt number	Entities protein name
**(1) Loss of Function of FBXW7 in Cancer and NOTCH1 Signaling**	3/6	3.31E-05	0.012	P62877<br>Q969H0-1<br>Q969H0-4	E3 ubiquitin-protein ligase *RBX1*<br>F-box/WD repeat-containing protein 7 *FBXW7* isoform 1<br>F-box/WD repeat-containing protein 7 *FBXW7* isoform 4
**(2) FBXW7 Mutants and NOTCH1 in Cancer**	3/6	3.31E-05	0.012	P62877<br>Q969H0-1<br>Q969H0-4	E3 ubiquitin-protein ligase *RBX1*<br>F-box/WD repeat-containing protein 7 *FBXW7* isoform 1<br>F-box/WD repeat-containing protein 7 *FBXW7* isoform 4
**(3) NOTCH1 Intracellular Domain Regulates Transcription**	5/48	1.30E-04	0.019	P49336<br>P62877<br>Q969H0-1<br>Q969H0-4<br>Q92830	Cyclin-dependent kinase 8 *CDK8*<br>E3 ubiquitin-protein ligase *RBX1*<br>F-box/WD repeat-containing protein 7 *FBXW7* isoform 1<br>F-box/WD repeat-containing protein 7 *FBXW7* isoform 4<br>Histone acetyltransferase *KAT2A*
**(4) Regulation of TP53 Expression**	2/2	1.94E-04	0.019	P04637<br>O75626	Cellular tumour antigen *P53*<br>PR domain zinc finger protein 1 *PRDM1*
**(5) Defective Base Excision Repair Associated with MUTYH**	2/2	1.94E-04	0.019	Q9UIF7-3<br>Q9UIF7-6	Adenine DNA glycosylase *MUTYH* isoform 3<br>Adenine DNA glycosylase *MUTYH* isoform 6
**(6) Pyrophosphate hydrolysis**	2/2	1.94E-04	0.019	Q9H2U2<br>Q15181	Inorganic pyrophosphatase 2, mitochondrial *IPYR2*<br>Inorganic pyrophosphatase *IPYR*
**(7) Defective MUTYH substrate processing**	2/2	1.94E-04	0.019	Q9UIF7-3<br>Q9UIF7-6	Adenine DNA glycosylase *MUTYH* isoform 3<br>Adenine DNA glycosylase *MUTYH* isoform 6
**(8) TP53 Regulates Metabolic Genes**	6/88	2.70E-04	0.019	P42345<br>Q9UGJ0<br>P04637<br>P14854<br>P10599<br>Q9HCJ0	Serine/threonine-protein kinase mTOR *MTOR*<br>5'-AMP-activated protein kinase subunit gamma-2 *AAKG2*<br>Cellular tumour antigen *P53*<br>Cytochrome c oxidase subunit 6B1 *CX6B1*<br>Thioredoxin *THIO*<br>Trinucleotide repeat-containing gene 6C protein *TNR6C*
**(9) Constitutive Signaling by NOTCH1 HD+PEST Domain Mutants**	5/59	3.34E-04	0.019	P49336<br>P62877<br>Q969H0-1<br>Q969H0-4<br>Q92830	Cyclin-dependent kinase 8 *CDK8*<br>E3 ubiquitin-protein ligase *RBX1*<br>F-box/WD repeat-containing protein 7 *FBXW7* isoform 1<br>F-box/WD repeat-containing protein 7 *FBXW7* isoform 4<br>Histone acetyltransferase *KAT2A*

Pathway name	Entities found	Entities p-value	Entities FDR	UniProt number	Entities protein name
**(10) Signaling by NOTCH1 HD+PEST Domain Mutants in Cancer**	5/59	3.34E-04	0.019	P49336<br>P62877<br>Q969H0-1<br>Q969H0-4<br>Q92830	Cyclin-dependent kinase 8 *CDK8*<br>E3 ubiquitin-protein ligase *RBX1*<br>F-box/WD repeat-containing protein 7 *FBXW7* isoform 1<br>F-box/WD repeat-containing protein 7 *FBXW7* isoform 4<br>Histone acetyltransferase *KAT2A*
**(11) Signaling by NOTCH1 PEST Domain Mutants in Cancer**	5/59	3.34E-04	0.019	P49336<br>P62877<br>Q969H0-1<br>Q969H0-4<br>Q92830	Cyclin-dependent kinase 8 *CDK8*<br>E3 ubiquitin-protein ligase *RBX1*<br>F-box/WD repeat-containing protein 7 *FBXW7* isoform 1<br>F-box/WD repeat-containing protein 7 *FBXW7* isoform 4<br>Histone acetyltransferase *KAT2A*
**(12) Constitutive Signaling by NOTCH1 PEST Domain Mutants**	5/59	3.34E-04	0.019	P49336<br>P62877<br>Q969H0-1<br>Q969H0-4<br>Q92830	Cyclin-dependent kinase 8 *CDK8*<br>E3 ubiquitin-protein ligase *RBX1*<br>F-box/WD repeat-containing protein 7 *FBXW7* isoform 1<br>F-box/WD repeat-containing protein 7 *FBXW7* isoform 4<br>Histone acetyltransferase *KAT2A*
**(13) Signaling by NOTCH1 in Cancer**	5/59	3.34E-04	0.019	P49336<br>P62877<br>Q969H0-1<br>Q969H0-4<br>Q92830	Cyclin-dependent kinase 8 *CDK8*<br>E3 ubiquitin-protein ligase *RBX1*<br>F-box/WD repeat-containing protein 7 *FBXW7* isoform 1<br>F-box/WD repeat-containing protein 7 *FBXW7* isoform 4<br>Histone acetyltransferase *KAT2A*
**(14) Signaling by WNT**	10/299	8.48E-04	0.044	P25054<br>Q13237<br>P09497<br>P62877<br>P56545<br>Q9P219<br>P35913<br>Q8N474<br>O14641<br>Q9HCJ0	Adenomatous polyposis coli protein *APC*<br>cGMP-dependent protein kinase 2 *KGP2*<br>Clathrin light chain B *CLCB*<br>E3 ubiquitin-protein ligase *RBX1*<br>PC-terminal-binding protein 2 *CTBP2*<br>Protein Daple *DAPLE*<br>Rod cGMP-specific 3',5'-cyclic phosphodiesterase subunit beta *PDE6B*<br>Secreted frizzled-related protein 1 *SFRP1*<br>Segment polarity protein dishevelled homolog *DVL2*<br>Trinucleotide repeat-containing gene 6C protein *TNR6C*

Pathway name	Entities found	Entities p-value	Entities FDR	UniProt number	Entities protein name
**(15) Signaling by NOTCH1**	5/74	9.23E-04	0.045	P49336<br>P62877<br>Q969H0-1<br>Q969H0-4<br>Q92830	Cyclin-dependent kinase 8 *CDK8*<br>E3 ubiquitin-protein ligase *RBX1*<br>F-box/WD repeat-containing protein 7 *FBXW7* isoform 1<br>F-box/WD repeat-containing protein 7 *FBXW7* isoform 4<br>Histone acetyltransferase *KAT2A*
(16) Signaling by NOTCH	8/205	0.0011	0.049	P04637<br>P49336<br>P62877<br>Q969H0-1<br>Q969H0-4<br>Q92830<br>Q14186<br>Q9HCJ0	Cellular tumour antigen *P53*<br>Cyclin-dependent kinase 8 *CDK8*<br>E3 ubiquitin-protein ligase *RBX1*<br>F-box/WD repeat-containing protein 7 *FBXW7* isoform 1<br>F-box/WD repeat-containing protein 7 *FBXW7* isoform 4<br>Histone acetyltransferase *KAT2A*<br>Transcription factor Dp-1 *TFDP1*<br>Trinucleotide repeat-containing gene 6C protein *TNR6C*
(17) Negative regulation of TCF-dependent signaling by DVL-interacting proteins	2/5	0.0012	0.049	Q9P219<br>O14641	Protein Daple *DAPLE*<br>Segment polarity protein dishevelled homolog *DVL2*
(18) Activation of NOXA and translocation to mitochondria	2/5	0.0012	0.049	P04637<br>Q14186	Cellular tumour antigen *P53*<br>Transcription factor Dp-1 *TFDP1*
(19) Diseases of Base Excision Repair	2/7	0.0023	0.087	Q9UIF7-3<br>Q9UIF7-6	Adenine DNA glycosylase *MUTYH* isoform 3<br>Adenine DNA glycosylase *MUTYH* isoform 6
(20) Insulin processing	3/27	0.0026	0.091	P16870<br>Q12840<br>P33176	Carboxypeptidase E *CBPE*<br>Kinesin heavy chain isoform 5A *KIF5A*<br>Kinesin-1 heavy chain *KINH*
(21) Oxidative Stress Induced Senescence	5/94	0.0026	0.091	P04637<br>P01100<br>P10599<br>Q14186<br>Q9HCJ0	Cellular tumour antigen *P53*<br>Proto-oncogene c-Fos *FOS*<br>Thioredoxin *THIO*<br>Transcription factor Dp-1 *TFDP1*<br>Trinucleotide repeat-containing gene 6C protein *TNR6C*
(22) Pre-NOTCH Transcription and Translation	4/62	0.0036	0.113	P04637<br>Q92830<br>Q14186<br>Q9HCJ0	Cellular tumour antigen *P53*<br>Histone acetyltransferase *KAT2A*<br>Transcription factor Dp-1 *TFDP1*<br>Trinucleotide repeat-containing gene 6C protein *TNR6C*

Pathway name	Entities found	Entities p-value	Entities FDR	UniProt number	Entities protein name
(23) Activation of PUMA and translocation to mitochondria	2/9	0.0038	0.113	P04637<br>Q14186	Cellular tumour antigen *P53*<br>Transcription factor Dp-1 *TFDP1*
(24) Transcriptional Regulation by TP53	10/367	0.0038	0.113	Q9UGJ0<br>Q92851<br>P04637<br>P14854<br>O75626<br>P01100<br>P42345<br>P10599<br>Q14186<br>Q9HCJ0	5'-AMP-activated protein kinase subunit gamma-2 *AAKG2*<br>Caspase-10 *CASP10*<br>Cellular tumour antigen *p53*<br>Cytochrome c oxidase subunit 6B1 *CX6B1*<br>PR domain zinc finger protein 1 *PRDM1*<br>Proto-oncogene c-Fos *FOS*<br>Serine/threonine-protein kinase mTOR *MTOR*<br>Thioredoxin *THIO*<br>Transcription factor Dp-1 *TFDP1*<br>Trinucleotide repeat-containing gene 6C protein *TNR6C*
(25) FOXO-mediated transcription	4/66	0.0045	0.124	Q8N139<br>Q969P5<br>P23511<br>P10599	ATP-binding cassette sub-family A member 6 *ABCA6*<br>F-box only protein 32 *FBX32*<br>Nuclear transcription factor Y subunit alpha *NFYA*<br>Thioredoxin *THIO*

Table B – Reactome analysis of the EPE6 interactors selected with the Protein-Pathway method

Pathway name	Entities found	Entities p-value	Entities FDR	UniProt number	Entities protein name
(1) Signaling by Hippo	3/20	4.21E-04	0.144	O14641<br>Q9UDY2<br>Q9GZV5	Segment polarity protein disheveled homolog *DVL-2*<br>Tight junction protein *ZO-2*<br>WW domain-containing transcription regulator protein 1 *WWTR1*
(2) Post-chaperonin tubulin folding pathway	3/23	6.31E-04	0.144	Q13509<br>O75347<br>Q9BTW9	Tubulin beta-3 chain *TUBB3*<br>Tubulin-specific chaperone A *TBCA*<br>Tubulin-specific chaperone D *TBCD*
(3) Activated NTRK3 signals through PI3K	2/6	8.81E-04	0.144	P35568<br>P27986	Insulin receptor substrate 1 *IRS1*<br>Phosphatidylinositol 3-kinase regulatory subunit alpha *PIK3R1*
(4) RNA Polymerase III Transcription Initiation from Type 3 Promoter	3/28	0.0011	0.144	Q16533<br>Q5SXM2<br>A6H8Y1	snRNA-activating protein complex subunit 1 *SNAPC1*<br>snRNA-activating protein complex subunit 4 *SNAPC4*<br>Transcription factor TFIIIB component B'' homolog *BDP1*
(5) HDR through Homologous Recombination (HRR)	4/66	0.0013	0.144	Q99708<br>Q07864<br>P49959<br>P12004	DNA endonuclease *RBBP8*<br>DNA polymerase epsilon catalytic subunit *POLE*<br>Double strand break repair protein *MRE11*<br>Proliferating cell nuclear antigen *PCNA*
(6) Erythrocytes take up oxygen and release carbon dioxide	2/8	0.0016	0.144	P69905<br>P68871	Hemoglobin subunit alpha *HBA1*<br>Hemoglobin subunit beta *HBB*
(7) PI3K/AKT activation	2/9	0.0020	0.144	P35568<br>P27986	Insulin receptor substrate 1 *IRS1*<br>Phosphatidylinositol 3-kinase regulatory subunit alpha *PIK3R1*
(8) RNA Polymerase III Transcription Initiation	3/36	0.0023	0.144	Q16533<br>Q5SXM2<br>A6H8Y1	snRNA-activating protein complex subunit 1 *SNAPC1*<br>snRNA-activating protein complex subunit 4 *SNAPC4*<br>Transcription factor TFIIIB component B'' homolog *BDP1*
(9) HDR through MMEJ (alt-NHEJ)	2/10	0.0024	0.144	Q99708<br>P49959	DNA endonuclease *RBBP8*<br>Double strand break repair protein *MRE11*
(10) Signaling by MET	4/80	0.0027	0.144	Q8N307<br>P27986<br>Q6VN20<br>Q96B97	Mucin-20 *MUC20*<br>Phosphatidylinositol 3-kinase regulatory subunit alpha *PIK3R1*<br>Ran-binding protein 10 *RANBP10*<br>SH3 domain-containing kinase-binding protein 1 *SH3KBP1*

Pathway name	Entities found	Entities p-value	Entities FDR	UniProt number	Entities protein name
(11) Metabolism of proteins	25/2012	0.0028	0.144	O95786<br>P07550<br>P04637<br>Q8IWV2<br>Q13618<br>Q13217<br>Q9Y297<br>P02671<br>Q92696<br>Q6ZVT0<br>P05019<br>P15088<br>Q8N307<br>P23511<br>Q9UBK2<br>Q14435<br>P12004<br>Q9Y6M0<br>P10599<br>Q9P031<br>Q5JRA6<br>Q13509<br>Q9Y4R7<br>O75347<br>Q9BTW9	Antiviral innate immune response receptor RIG-I *DDX58*<br>Beta-2 adrenergic receptor *ADRB2*<br>Cellular tumour antigen *p53*<br>Contactin-4 *CNTN4*<br>Cullin-3 *CUL3*<br>DnaJ homolog subfamily C member 3 *DNAJC3*<br>F-box/WD repeat-containing protein 1A *BTRC*<br>Fibrinogen alpha chain *FGA*<br>Geranylgeranyl transferase type-2 subunit alpha *RABGGATA*<br>Inactive polyglycylase *TTLL10*<br>Insulin-like growth factor I *IGF1*<br>Mast cell carboxypeptidase A *CPA3*<br>Mucin-20 *MUC20*<br>Nuclear transcription factor Y subunit alpha *NFYA*<br>Peroxisome proliferator-activated receptor gamma coactivator 1-alpha *PPARGC1A*<br>Polypeptide N-acetylgalactosaminyltransferase 3 *GLNT3*<br>Proliferating cell nuclear antigen *PCNA*<br>Testisin *PRSS21*<br>Thioredoxin *THIO*<br>Thyroid transcription factor 1-associated protein 26 *CCDC59*<br>Transport and Golgi organization protein 1 homolog *MIA3*<br>Tubulin beta-3 chain *TUBB3*<br>Tubulin monoglycylase *TTLL3*<br>Tubulin-specific chaperone A *TBCA*<br>Tubulin-specific chaperone D *TBCD*
(12) RNA Polymerase III Abortive and Retractive Initiation	3/41	0.0033	0.144	Q16533<br>Q5SXM2<br>A6H8Y1	snRNA-activating protein complex subunit 1 *SNAPC1*<br>snRNA-activating protein complex subunit 4 *SNAPC4*<br>Transcription factor TFIIIB component B'' homolog *BDP1*
(13) RNA Polymerase III Transcription	3/41	0.0033	0.144	Q16533<br>Q5SXM2<br>A6H8Y1	snRNA-activating protein complex subunit 1 *SNAPC1*<br>snRNA-activating protein complex subunit 4 *SNAPC4*<br>Transcription factor TFIIIB component B'' homolog *BDP1*
(14) Erythropoietin activates Phosphoinositide-3-kinase (PI3K)	2/12	0.0034	0.144	P27986<br>P48736	Phosphatidylinositol 3-kinase regulatory subunit alpha *PIK3R1*<br>Thyroid transcription factor 1-associated protein 26 *CCDC59*

Pathway name	Entities found	Entities p-value	Entities FDR	UniProt number	Entities protein name
(15) MET activates RAS signaling	2/12	0.0034	0.144	Q8N307<br>Q6VN20	Mucin-20 *MUC20*<br>Ran-binding protein 10 *RANBP10*
(16) Erythrocytes take up carbon dioxide and release oxygen	2/12	0.0034	0.144	P69905<br>P68871	Hemoglobin subunit alpha *HBA1*<br>Hemoglobin subunit beta *HBB*
(17) O2/CO2 exchange in erythrocytes	2/12	0.0034	0.144	P69905<br>P68871	Hemoglobin subunit alpha *HBA1*<br>Hemoglobin subunit beta *HBB*
(18) Carboxyterminal post-translational modifications of tubulin	3/43	0.0037	0.145	Q6ZVT0<br>Q13509<br>Q9Y4R7	Inactive polyglycylase *TTLL10*<br>Tubulin beta-3 chain *TUBB3*<br>Tubulin monoglycylase *TTLL3*
(19) DNA Double-Strand Break Repair	5/148	0.0043	0.146	P04637<br>Q99708<br>Q07864<br>P49959<br>P12004	Cellular tumour antigen *p53*<br>DNA endonuclease *RBBP8*<br>DNA polymerase epsilon catalytic subunit *POLE*<br>Double strand break repair protein *MRE11*<br>Proliferating cell nuclear antigen *PCNA*
(20) TP53 regulates transcription of several additional cell death genes whose specific roles in p53-dependent apoptosis remain uncertain	2/14	0.0046	0.146	P04637<br>Q92696	Cellular tumour antigen *p53*<br>Geranylgeranyl transferase type-2 subunit alpha *RABGGATA*
(21) SEMA3A-Plexin repulsion signaling by inhibiting Integrin adhesion	2/14	0.0046	0.146	O75051<br>Q14563	Plexin-A2 *PLXNA2*<br>Semaphorin-3A *SEMA3*
(22) Transcriptional Regulation by TP53	8/367	0.0047	0.146	P04637<br>P50750<br>P14854<br>Q99708<br>P49959<br>Q92696<br>P12004<br>P10599	Cellular tumour antigen *p53*<br>Cyclin-dependent kinase 9 *CDK9*<br>Cytochrome c oxidase subunit 6B1 *COX6B1*<br>DNA endonuclease *RBBP8*<br>Double strand break repair protein *MRE11*<br>Geranylgeranyl transferase type-2 subunit alpha *RABGGATA*<br>Proliferating cell nuclear antigen *PCNA*<br>Thioredoxin *THIO*
(23) Toll-like Receptor Cascades	5/156	0.0053	0.146	Q9Y297<br>P02671<br>Q9NWZ3<br>Q13233<br>Q9BT09	F-box/WD repeat-containing protein 1A *BTRC*<br>Fibrinogen alpha chain *FGA*<br>Interleukin-1 receptor-associated kinase 4 *IRAK4*<br>Mitogen-activated protein kinase kinase kinase 1 *MAP3K1*<br>Protein canopy homolog 3 *CNPY3*

Pathway name	Entities found	Entities p-value	Entities FDR	UniProt number	Entities protein name
(24) Signaling by EGFR	3/51	0.0060	0.146	O43184<br>P27986<br>Q96B97	Disintegrin and metalloproteinase domain-containing protein 12 *ADAM12*<br>Phosphatidylinositol 3-kinase regulatory subunit alpha *PIK3R1*<br>SH3 domain-containing kinase-binding protein 1 *SH3KBP1*
(25) CRMPs in Sema3A signaling	2/16	0.0060	0.146	O75051<br>Q14563	Plexin-A2 *PLXNA2*<br>Semaphorin-3A *SEMA3*

Table C – **Reactome analysis of the AAE6 and EPE6 overlapping interactors selected with the Protein-Pathway method**

Pathway name	Entities found	Entities p-value	Entities FDR	UniProt number	Entities protein name
(1) TP53 Regulates Metabolic Genes	3/88	0.0005	0.094	P04637<br>P14854<br>P10599	Cellular tumour antigen *P53*<br>Cytochrome c oxidase subunit 6B1 *CX6B1*<br>Thioredoxin *THIO*
(2) Late endosomal microautophagy	2/34	0.0016	0.094	Q9H444<br>P68871	Charged multivesicular body protein 4b CHMP4B<br>Hemoglobin subunit beta HBB
(3) Carboxyterminal post-translational modifications of tubulin	2/43	0.0026	0.094	Q6ZVT0<br>Q9Y4R7	Inactive polyglycylase *TTLL10*<br>Tubulin monoglycylase *TTLL3*
(4) Regulation of TP53 Expression	1/2	0.0035	0.094	P04637	Cellular tumour antigen *P53*
(5) FOXO-mediated transcription	2/66	0.0059	0.094	P23511<br>P10599	Nuclear transcription factor Y subunit alpha *NFYA*<br>Thioredoxin *THIO*
(6) Transcriptional activation of cell cycle inhibitor p21	1/4	0.0070	0.094	P04637	Cellular tumour antigen *P53*
(7) Transcriptional activation of p53 responsive genes	1/4	0.0070	0.094	P04637	Cellular tumour antigen *P53*
(8) Negative regulation of TCF-dependent signaling by DVL-interacting proteins	1/5	0.0087	0.094	O14641	Segment polarity protein dishevelled homolog *DVL2*
(9) Reelin signalling pathway	1/5	0.0087	0.094	Q96B97	SH3 domain-containing kinase-binding protein 1 *SH3KBP1*
(10) Activation of NOXA and translocation to mitochondria	1/5	0.0087	0.094	P04637	Cellular tumour antigen *P53*
(11) Protein repair	1/6	0.0105	0.094	P10599	Thioredoxin *THIO*
(12) Oxidative Stress Induced Senescence	2/94	0.0117	0.094	P04637<br>P10599	Cellular tumour antigen *P53*<br>Thioredoxin *THIO*
(13) RUNX3 regulates CDKN1A transcription	1/7	0.0122	0.094	P04637	Cellular tumour antigen *P53*
(14) Cell Cycle, Mitotic	4/536	0.0129	0.094	P04637<br>Q13352<br>Q9H444<br>Q96GY3	Cellular tumour antigen P53<br>Centromere protein R CENPR<br>Charged multivesicular body protein 4b *CHMP4B*<br>Protein lin-37 homolog LIN37
(15) WNT mediated activation of DVL	1/8	0.0139	0.094	O14641	Segment polarity protein dishevelled homolog *DVL2*

Pathway name	Entities found	Entities p-value	Entities FDR	UniProt number	Entities protein name
(16) Erythrocytes take up oxygen and release carbon dioxide	1/8	0.0139	0.094	P68871	Hemoglobin subunit beta *HBB*
(17) Cargo recognition for clathrin-mediated endocytosis	2/105	0.0145	0.094	O14641<br>Q96B97	Segment polarity protein dishevelled homolog DVL2<br>SH3 domain-containing kinase-binding protein 1 SH3KBP1
(18) Activation of PUMA and translocation to mitochondria	1/9	0.0157	0.094	P04637	Cellular tumour antigen *P53*
(19) PI5P Regulates TP53 Acetylation	1/9	0.0157	0.094	P04637	Cellular tumour antigen *P53*
(20) Metabolism of proteins	8/2012	0.0158	0.094	P04637<br>P02671<br>Q6ZVT0<br>P23511<br>Q9Y6M0<br>P10599<br>Q9P031<br>Q9Y4R7	Cellular tumour antigen P53<br>Fibrinogen alpha chain FGA<br>Inactive polyglycylase TTLL10<br>Nuclear transcription factor Y subunit alpha NFYA<br>Testisin PRSS21<br>Thioredoxin THIO<br>Thyroid transcription factor 1-associated protein 26 CCDC59<br>Tubulin monoglycylase TTLL3
(21) ATF6 (ATF6-alpha) activates chaperone genes	1/10	0.0174	0.094	P23511	Nuclear transcription factor Y subunit alpha *NFYA*
(22) Regulation of FOXO transcriptional activity by acetylation	1/10	0.0174	0.094	P10599	Thioredoxin *THIO*
(23) TP53 Regulates Transcription of Death Receptors and Ligands	1/12	0.0209	0.094	P04637	Cellular tumour antigen *P53*
(24) TP53 Regulates Transcription of Caspase Activators and Caspases	1/12	0.0209	0.094	P04637	Cellular tumour antigen *P53*
(25) WNT5:FZD7-mediated leishmania damping	1/12	0.0209	0.094	O14641	Segment polarity protein dishevelled homolog *DVL2*
